# 5bα,6,7,13bα,14,15-Hexahydro­acridino[4,3-*c*]acridine

**DOI:** 10.1107/S1600536808014803

**Published:** 2008-05-21

**Authors:** Jason Ashmore, Roger Bishop, Donald C. Craig, Marcia L. Scudder

**Affiliations:** a School of Chemistry, University of New South Wales, Sydney 2052, Australia

## Abstract

The racemic title compound, C_24_H_20_N_2_, gives spontaneous resolution with the formation of conglomerate crystals in the space group *P*2_1_2_1_2_1_ when crystallized from ethyl acetate. The twisted mol­ecules pack in parallel regions (*ab* plane) which then form a herringbone pattern along *c*.

## Related literature

Condensation of two equivalents of 2-amino­benzaldehyde with one of *cis*-bicyclo­[4.4.0]decane-2,7-dione affords the title compound by means of Friedländer condensation (Cheng & Yan, 1982 ▶). Substituted derivatives of mol­ecules of this general V-shaped type frequently show inclusion properties (Bishop, 2006 ▶). For related literature, see: Collet *et al.* (1980 ▶); Jacques *et al.* (1981 ▶); Marjo *et al.* (1997 ▶); Peet & Cargill (1973 ▶); Smith & Opie (1955 ▶).
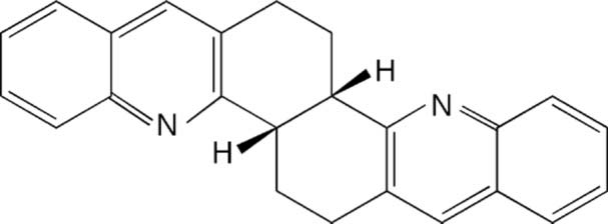


## Experimental

### 

#### Crystal data

C_24_H_20_N_2_
*M* *_r_* = 336.4
Orthorhombic, 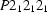

*a* = 8.863 (3) Å
*b* = 9.759 (4) Å
*c* = 20.071 (8) Å
*V* = 1736 (1) Å^3^
*Z* = 4
Mo *K*α radiation
μ = 0.07 mm^−1^
*T* = 294 K
0.29 × 0.27 × 0.03 mm

#### Data collection

Enraf–Nonius CAD-4 diffractometer
Absorption correction: none
1100 measured reflections
1100 independent reflections
737 reflections with *I* > 2σ(*I*)
θ_max_ = 21°
1 standard reflection frequency: 30 min intensity decay: none

#### Refinement

*R*[*F* ^2^ > 2σ(*F* ^2^)] = 0.048
*wR*(*F* ^2^) = 0.059
*S* = 1.64
1100 reflections
94 parameters
H-atom parameters constrained
Δρ_max_ = 0.26 e Å^−3^
Δρ_min_ = −0.42 e Å^−3^

### 

Data collection: *CAD-4 Manual* (Schagen *et al.*, 1989 ▶); cell refinement: *CAD-4 Manual*; data reduction: local program; program(s) used to solve structure: *SIR92* (Altomare *et al.*, 1994 ▶); program(s) used to refine structure: *RAELS* (Rae, 2000 ▶); molecular graphics: *ORTEPII* (Johnson, 1976 ▶) and *CrystalMaker* (CrystalMaker, 2005 ▶); software used to prepare material for publication: local programs.

## Supplementary Material

Crystal structure: contains datablocks global, I. DOI: 10.1107/S1600536808014803/bq2079sup1.cif

Structure factors: contains datablocks I. DOI: 10.1107/S1600536808014803/bq2079Isup2.hkl

Additional supplementary materials:  crystallographic information; 3D view; checkCIF report

## Figures and Tables

**Fig. 1.**
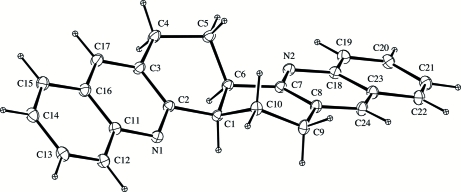
Molecular structure of the compound, with ellipsoids drawn at 30% probability level.

**Fig. 2.**
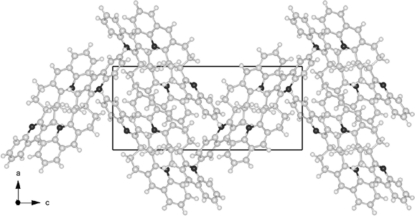
Cell diagram showing the parallel regions (in the ab plane) which pack in a herringbone pattern.

## supplementary crystallographic information

### Comment

The title compound was prepared as racemic material by Friedländer condensation (Cheng & Yan, 1982), but the crystallization process resulted in self-resolution and formation of a conglomerate (Collet *et al.*, 1980; Jacques *et al.*, 1981) (Fig 1). The two aromatic extremities of the molecule are essentially planar but are not coplanar, instead they exhibit a relative twist with the angle between the normals to the planes of 29.5 (2)°. These awkwardly shaped molecules pack in parallel regions in the *ab* plane. These regions then interact in herringbone fashion along *c* (Fig 2). Within the *ab* plane, molecules take part in edge-face aromatic interactions with H···π distance of about 3.4 Å. Because of the twisted nature of the molecule, it is not possible for them to take part in edge-edge C—H···N interactions that we have previously observed (Marjo *et al.*, 1997). The crystals do not exhibit solvent inclusion, in contrast to other derivatives, which are V-shaped (Bishop, 2006).

### Experimental

Racemic *cis*-bicyclo[4.4.0]decane-2,7-dione (Peet & Cargill, 1973) (0.54 g, 3.25 mmol) and 2-aminobenzaldehyde (Smith & Opie, 1955) (0.88 g, 7.26 mmol) were dissolved in methanol (15 mL) with heating. To the cooled solution was added sodium hydroxide solution (2M; 2.5 mL) and the mixture stirred at rt for 2 days. The solid precipitate was filtered, and then recrystallised from ethyl acetate to yield the title compound (0.63 g, 58%) as pale yellow plates. ^13^C NMR (75.5 MHz, CDCl_3_) δ: 27.9 (CH_2_), 29.5 (CH_2_), 42.7 (CH), 126.2 (CH), 127.3 (CH), 127.6 (C), 128.7 (CH), 128.9 (CH), 130.4 (C), 135.6 (CH), 147.5 (C), 161.4 (C); ^1^H NMR (300 MHz, CDCl_3_) δ: 2.09-2.23 (m, 2H), 2.45-2.50 (m, 2H), 3.07-3.16 (m, 2H), 3.23-3.34 (m, 2H), 3.70 (d, *J* = 9.6 Hz, 2H), 7.44-7.49 (m, 2H), 7.61-7.65 (m, 2H), 7.74 (d, *J* = 8.3 Hz, 2H), 7.86-7.90 (m, 2H), 8.05 (d, *J* = 8.7 Hz, 2H). X-ray quality crystals were obtained from ethyl acetate solution. The identical product is obtained if *trans*-bicyclo[4.4.0]decane-2,7-dione is used but the reaction takes longer.

### Refinement

Hydrogen atoms attached to C were included at calculated positions (C—H = 1.0 Å) and were refined with isotropic thermal parameters equivalent to those of the atom to which they were bonded.

### Crystal data

**Table:**

C_24_H_20_N_2_	*D*_x_ = 1.29 Mg m^−^^3^
*M**_r_* = 336.4	Mo *K*α radiation λ = 0.71073 Å
Orthorhombic, *P*2_1_2_1_2_1_	Cell parameters from 11 reflections
*a* = 8.863 (3) Å	θ = 10–11º
*b* = 9.759 (4) Å	µ = 0.08 mm^−^^1^
*c* = 20.071 (8) Å	*T* = 294 K
*V* = 1736 (1) Å^3^	Plate, colourless
*Z* = 4	0.29 × 0.27 × 0.03 mm
*F*_000_ = 712.0	

### Data collection

**Table:**

Enraf–Nonius CAD-4 diffractometer	*h* = 0→8
ω–2θ scans	*k* = 0→9
Absorption correction: none	*l* = 0→20
1100 measured reflections	1 standard reflections
1100 independent reflections	every 30 min
737 reflections with *I* > 2σ(*I*)	intensity decay: none
θ_max_ = 21º	

### Refinement

**Table:**

Refinement on *F*	H-atom parameters constrained
*R*[*F*^2^ > 2σ(*F*^2^)] = 0.048	*w* = 1/[σ^2^(*F*) + 0.0004*F*^2^]
*wR*(*F*^2^) = 0.059	(Δ/σ)_max_ = 0.001
*S* = 1.64	Δρ_max_ = 0.26 e Å^−^^3^
1100 reflections	Δρ_min_ = −0.42 e Å^−^^3^
94 parameters	Extinction correction: none

### Fractional atomic coordinates and isotropic or equivalent isotropic displacement parameters (Å^2^)

**Table:**

	*x*	*y*	*z*	*U*_iso_*/*U*_eq_	
N1	−0.2254 (5)	0.4393 (4)	0.4278 (2)	0.0464 (9)	
N2	0.2369 (5)	0.5930 (4)	0.2179 (2)	0.0470 (7)	
C1	−0.0205 (6)	0.4796 (5)	0.3538 (3)	0.0427 (9)	
C2	−0.1687 (6)	0.5253 (6)	0.3840 (3)	0.0442 (8)	
C3	−0.2383 (6)	0.6509 (6)	0.3667 (3)	0.0517 (6)	
C4	−0.1694 (6)	0.7391 (5)	0.3130 (3)	0.060 (1)	
C5	0.0008 (6)	0.7137 (5)	0.3058 (3)	0.0535 (6)	
C6	0.0323 (6)	0.5681 (5)	0.2944 (3)	0.0438 (8)	
C7	0.1968 (6)	0.5402 (5)	0.2756 (3)	0.0435 (8)	
C8	0.2927 (6)	0.4617 (6)	0.3160 (3)	0.0478 (8)	
C9	0.2386 (7)	0.3999 (6)	0.3811 (3)	0.055 (1)	
C10	0.0995 (6)	0.4728 (6)	0.4075 (2)	0.0500 (9)	
C11	−0.3538 (6)	0.4784 (6)	0.4617 (3)	0.0491 (9)	
C12	−0.4120 (7)	0.3867 (6)	0.5090 (3)	0.055 (1)	
C13	−0.5368 (6)	0.4207 (6)	0.5461 (3)	0.059 (1)	
C14	−0.6080 (7)	0.5469 (6)	0.5353 (3)	0.061 (1)	
C15	−0.5551 (7)	0.6374 (6)	0.4883 (3)	0.062 (1)	
C16	−0.4254 (7)	0.6036 (5)	0.4505 (3)	0.0541 (7)	
C17	−0.3670 (7)	0.6883 (6)	0.4001 (3)	0.0587 (9)	
C18	0.3795 (6)	0.5649 (5)	0.1950 (3)	0.0481 (7)	
C19	0.4207 (7)	0.6142 (6)	0.1309 (3)	0.0553 (9)	
C20	0.5578 (7)	0.5824 (6)	0.1045 (3)	0.058 (1)	
C21	0.6593 (6)	0.4985 (6)	0.1403 (3)	0.057 (1)	
C22	0.6250 (7)	0.4515 (6)	0.2028 (3)	0.057 (1)	
C23	0.4821 (6)	0.4854 (6)	0.2310 (3)	0.0502 (8)	
C24	0.4353 (6)	0.4356 (6)	0.2941 (3)	0.053 (1)	
HC1	−0.0355	0.3843	0.3367	0.045	
H1C4	−0.1862	0.8376	0.3246	0.074	
H2C4	−0.2198	0.7180	0.2696	0.065	
H1C5	0.0530	0.7439	0.3474	0.057	
H2C5	0.0397	0.7679	0.2671	0.061	
HC6	−0.0302	0.5398	0.2553	0.047	
H1C9	0.3212	0.4072	0.4149	0.066	
H2C9	0.2136	0.3011	0.3736	0.060	
H1C10	0.1275	0.5679	0.4214	0.054	
H2C10	0.0591	0.4215	0.4468	0.056	
HC12	−0.3622	0.2958	0.5159	0.062	
HC13	−0.5765	0.3559	0.5804	0.066	
HC14	−0.6987	0.5716	0.5624	0.067	
HC15	−0.6080	0.7266	0.4808	0.073	
HC17	−0.4193	0.7760	0.3886	0.072	
HC19	0.3487	0.6726	0.1051	0.064	
HC20	0.5867	0.6184	0.0596	0.065	
HC21	0.7581	0.4731	0.1197	0.062	
HC22	0.6988	0.3943	0.2282	0.066	
HC24	0.5066	0.3815	0.3224	0.065	

### Atomic displacement parameters (Å^2^)

**Table:**

	*U*^11^	*U*^22^	*U*^33^	*U*^12^	*U*^13^	*U*^23^
N1	0.0488 (6)	0.048 (1)	0.0427 (8)	0.0013 (6)	0.0019 (6)	0.0061 (6)
N2	0.0502 (7)	0.047 (1)	0.0444 (9)	0.0024 (6)	0.0029 (7)	0.0088 (7)
C1	0.0464 (7)	0.041 (1)	0.0409 (9)	0.0038 (7)	0.0000 (6)	0.0070 (8)
C2	0.0470 (7)	0.043 (1)	0.0429 (9)	0.0032 (6)	0.0012 (7)	0.0052 (7)
C3	0.0523 (7)	0.0458 (8)	0.057 (1)	0.0085 (8)	0.0076 (7)	0.0085 (9)
C4	0.061 (1)	0.049 (1)	0.072 (2)	0.015 (1)	0.015 (1)	0.019 (2)
C5	0.058 (1)	0.0405 (9)	0.062 (1)	0.0067 (8)	0.0120 (9)	0.0108 (9)
C6	0.0476 (7)	0.041 (1)	0.0431 (9)	0.0047 (7)	0.0011 (7)	0.0084 (8)
C7	0.0470 (7)	0.042 (1)	0.0418 (9)	0.0028 (6)	0.0007 (7)	0.0061 (7)
C8	0.0464 (7)	0.053 (1)	0.0440 (8)	0.0058 (6)	0.0002 (6)	0.0085 (7)
C9	0.0490 (7)	0.068 (2)	0.0476 (9)	0.0108 (8)	0.0007 (7)	0.0178 (8)
C10	0.0475 (7)	0.061 (2)	0.041 (1)	0.0037 (7)	−0.0006 (7)	0.0095 (7)
C11	0.0493 (7)	0.054 (1)	0.0445 (9)	−0.0010 (6)	0.0033 (6)	0.0019 (6)
C12	0.0547 (9)	0.064 (2)	0.047 (1)	−0.0039 (9)	0.0065 (9)	0.0055 (8)
C13	0.055 (1)	0.074 (2)	0.049 (1)	−0.008 (1)	0.008 (1)	−0.001 (1)
C14	0.053 (1)	0.073 (2)	0.056 (2)	−0.006 (1)	0.010 (1)	−0.010 (1)
C15	0.055 (1)	0.065 (2)	0.067 (2)	0.002 (1)	0.014 (1)	−0.005 (1)
C16	0.0513 (8)	0.0549 (9)	0.056 (1)	0.0030 (8)	0.0080 (8)	−0.0006 (9)
C17	0.0559 (9)	0.0520 (9)	0.068 (2)	0.011 (1)	0.013 (1)	0.007 (1)
C18	0.0497 (7)	0.050 (1)	0.0442 (9)	−0.0004 (6)	0.0032 (7)	0.0044 (6)
C19	0.055 (1)	0.064 (2)	0.047 (1)	−0.0023 (9)	0.006 (1)	0.009 (1)
C20	0.055 (1)	0.070 (2)	0.048 (1)	−0.007 (1)	0.007 (1)	0.001 (1)
C21	0.0507 (8)	0.068 (2)	0.052 (1)	−0.0038 (8)	0.0070 (9)	−0.005 (1)
C22	0.0484 (7)	0.068 (2)	0.054 (1)	0.0035 (7)	0.0051 (7)	0.001 (1)
C23	0.0473 (7)	0.056 (1)	0.0470 (8)	0.0025 (6)	0.0025 (6)	0.0027 (7)
C24	0.0471 (7)	0.064 (2)	0.0490 (8)	0.0082 (6)	0.0015 (6)	0.0097 (7)

### Geometric parameters (Å, °)

**Table:**

N1—C2	1.316 (6)	C10—H2C10	1.000
N1—C11	1.378 (6)	C11—C12	1.404 (7)
N2—C7	1.316 (6)	C11—C16	1.395 (7)
N2—C18	1.373 (6)	C12—C13	1.373 (7)
C1—C2	1.514 (7)	C12—HC12	1.000
C1—C6	1.544 (7)	C13—C14	1.400 (8)
C1—C10	1.516 (6)	C13—HC13	1.000
C1—HC1	1.000	C14—C15	1.376 (7)
C2—C3	1.415 (7)	C14—HC14	1.000
C3—C4	1.508 (7)	C15—C16	1.417 (7)
C3—C17	1.373 (7)	C15—HC15	1.000
C4—C5	1.536 (7)	C16—C17	1.405 (7)
C4—H1C4	1.000	C17—HC17	1.000
C4—H2C4	1.000	C18—C19	1.422 (7)
C5—C6	1.466 (7)	C18—C23	1.397 (7)
C5—H1C5	1.000	C19—C20	1.362 (7)
C5—H2C5	1.000	C19—HC19	1.000
C6—C7	1.531 (7)	C20—C21	1.413 (7)
C6—HC6	1.000	C20—HC20	1.000
C7—C8	1.403 (6)	C21—C22	1.370 (7)
C8—C9	1.517 (7)	C21—HC21	1.000
C8—C24	1.362 (7)	C22—C23	1.426 (7)
C9—C10	1.519 (8)	C22—HC22	1.000
C9—H1C9	1.000	C23—C24	1.418 (7)
C9—H2C9	1.000	C24—HC24	1.000
C10—H1C10	1.000		
			
C2—N1—C11	117.9 (5)	C1—C10—H2C10	109.3
C7—N2—C18	117.7 (5)	C9—C10—H1C10	109.3
C2—C1—C6	114.0 (4)	C9—C10—H2C10	109.3
C2—C1—C10	109.7 (4)	H1C10—C10—H2C10	109.5
C2—C1—HC1	107.2	N1—C11—C12	117.4 (5)
C6—C1—C10	111.1 (4)	N1—C11—C16	122.5 (5)
C6—C1—HC1	107.2	C12—C11—C16	120.0 (5)
C10—C1—HC1	107.2	C11—C12—C13	120.5 (6)
N1—C2—C1	114.3 (5)	C11—C12—HC12	119.7
N1—C2—C3	123.3 (5)	C13—C12—HC12	119.7
C1—C2—C3	122.3 (5)	C12—C13—C14	119.5 (6)
C2—C3—C4	119.6 (5)	C12—C13—HC13	120.2
C2—C3—C17	118.2 (5)	C14—C13—HC13	120.2
C4—C3—C17	122.2 (5)	C13—C14—C15	121.1 (5)
C3—C4—C5	111.9 (5)	C13—C14—HC14	119.4
C3—C4—H1C4	108.8	C15—C14—HC14	119.4
C3—C4—H2C4	108.8	C14—C15—C16	119.6 (6)
C5—C4—H1C4	108.8	C14—C15—HC15	120.2
C5—C4—H2C4	108.8	C16—C15—HC15	120.2
H1C4—C4—H2C4	109.5	C11—C16—C15	119.1 (5)
C4—C5—C6	110.9 (5)	C11—C16—C17	117.7 (5)
C4—C5—H1C5	109.1	C15—C16—C17	123.2 (5)
C4—C5—H2C5	109.1	C3—C17—C16	120.1 (5)
C6—C5—H1C5	109.1	C3—C17—HC17	120.0
C6—C5—H2C5	109.1	C16—C17—HC17	120.0
H1C5—C5—H2C5	109.5	N2—C18—C19	118.1 (5)
C1—C6—C5	111.4 (5)	N2—C18—C23	122.5 (5)
C1—C6—C7	112.3 (4)	C19—C18—C23	119.3 (6)
C1—C6—HC6	106.5	C18—C19—C20	120.3 (6)
C5—C6—C7	113.0 (5)	C18—C19—HC19	119.8
C5—C6—HC6	106.5	C20—C19—HC19	119.8
C7—C6—HC6	106.5	C19—C20—C21	120.1 (5)
N2—C7—C6	113.9 (5)	C19—C20—HC20	119.9
N2—C7—C8	124.0 (5)	C21—C20—HC20	119.9
C6—C7—C8	122.1 (5)	C20—C21—C22	121.2 (6)
C7—C8—C9	121.6 (5)	C20—C21—HC21	119.4
C7—C8—C24	118.6 (5)	C22—C21—HC21	119.4
C9—C8—C24	119.8 (5)	C21—C22—C23	118.9 (6)
C8—C9—C10	111.8 (5)	C21—C22—HC22	120.5
C8—C9—H1C9	108.9	C23—C22—HC22	120.5
C8—C9—H2C9	108.9	C18—C23—C22	120.1 (5)
C10—C9—H1C9	108.9	C18—C23—C24	117.5 (5)
C10—C9—H2C9	108.9	C22—C23—C24	122.4 (5)
H1C9—C9—H2C9	109.5	C8—C24—C23	119.7 (5)
C1—C10—C9	110.0 (4)	C8—C24—HC24	120.2
C1—C10—H1C10	109.3	C23—C24—HC24	120.2
			
C11—N1—C2—C1	174.9 (4)	C7—C8—C9—H1C9	−141.1
C11—N1—C2—C3	−4.6 (7)	C7—C8—C9—H2C9	99.6
C2—N1—C11—C12	−179.0 (5)	C24—C8—C9—C10	162.6 (5)
C2—N1—C11—C16	0.9 (7)	C24—C8—C9—H1C9	42.3
C18—N2—C7—C6	−175.6 (4)	C24—C8—C9—H2C9	−77.1
C18—N2—C7—C8	2.6 (7)	C7—C8—C24—C23	−1.2 (8)
C7—N2—C18—C19	176.1 (5)	C7—C8—C24—HC24	178.8
C7—N2—C18—C23	−1.1 (7)	C9—C8—C24—C23	175.6 (5)
C6—C1—C2—N1	170.4 (4)	C9—C8—C24—HC24	−4.4
C6—C1—C2—C3	−10.1 (7)	C8—C9—C10—C1	52.2 (6)
C10—C1—C2—N1	−64.2 (6)	C8—C9—C10—H1C10	−67.9
C10—C1—C2—C3	115.2 (6)	C8—C9—C10—H2C10	172.3
HC1—C1—C2—N1	51.9	H1C9—C9—C10—C1	172.6
HC1—C1—C2—C3	−128.6	H1C9—C9—C10—H1C10	52.5
C2—C1—C6—C5	38.9 (6)	H1C9—C9—C10—H2C10	−67.4
C2—C1—C6—C7	166.9 (4)	H2C9—C9—C10—C1	−68.1
C2—C1—C6—HC6	−76.8	H2C9—C9—C10—H1C10	171.8
C10—C1—C6—C5	−85.6 (6)	H2C9—C9—C10—H2C10	52.0
C10—C1—C6—C7	42.4 (6)	N1—C11—C12—C13	178.0 (5)
C10—C1—C6—HC6	158.6	N1—C11—C12—HC12	−2.0
HC1—C1—C6—C5	157.5	C16—C11—C12—C13	−1.9 (8)
HC1—C1—C6—C7	−74.5	C16—C11—C12—HC12	178.1
HC1—C1—C6—HC6	41.7	N1—C11—C16—C15	−178.7 (5)
C2—C1—C10—C9	168.5 (5)	N1—C11—C16—C17	3.4 (8)
C2—C1—C10—H1C10	−71.5	C12—C11—C16—C15	1.2 (8)
C2—C1—C10—H2C10	48.4	C12—C11—C16—C17	−176.7 (5)
C6—C1—C10—C9	−64.6 (6)	C11—C12—C13—C14	1.3 (8)
C6—C1—C10—H1C10	55.5	C11—C12—C13—HC13	−178.7
C6—C1—C10—H2C10	175.3	HC12—C12—C13—C14	−178.7
HC1—C1—C10—C9	52.3	HC12—C12—C13—HC13	1.3
HC1—C1—C10—H1C10	172.4	C12—C13—C14—C15	0.1 (9)
HC1—C1—C10—H2C10	−67.7	C12—C13—C14—HC14	−179.9
N1—C2—C3—C4	−176.5 (5)	HC13—C13—C14—C15	−179.9
N1—C2—C3—C17	3.9 (8)	HC13—C13—C14—HC14	0.1
C1—C2—C3—C4	4.1 (8)	C13—C14—C15—C16	−0.8 (9)
C1—C2—C3—C17	−175.5 (5)	C13—C14—C15—HC15	179.2
C2—C3—C4—C5	−25.4 (7)	HC14—C14—C15—C16	179.2
C2—C3—C4—H1C4	−145.7	HC14—C14—C15—HC15	−0.8
C2—C3—C4—H2C4	95.0	C14—C15—C16—C11	0.2 (8)
C17—C3—C4—C5	154.2 (6)	C14—C15—C16—C17	178.0 (5)
C17—C3—C4—H1C4	33.8	HC15—C15—C16—C11	−179.8
C17—C3—C4—H2C4	−85.5	HC15—C15—C16—C17	−2.0
C2—C3—C17—C16	0.6 (8)	C11—C16—C17—C3	−4.0 (8)
C2—C3—C17—HC17	−179.4	C11—C16—C17—HC17	176.0
C4—C3—C17—C16	−179.0 (5)	C15—C16—C17—C3	178.2 (6)
C4—C3—C17—HC17	1.0	C15—C16—C17—HC17	−1.8
C3—C4—C5—C6	54.8 (7)	N2—C18—C19—C20	−176.2 (5)
C3—C4—C5—H1C5	−65.5	N2—C18—C19—HC19	3.8
C3—C4—C5—H2C5	175.0	C23—C18—C19—C20	1.0 (9)
H1C4—C4—C5—C6	175.1	C23—C18—C19—HC19	−179.0
H1C4—C4—C5—H1C5	54.9	N2—C18—C23—C22	175.6 (5)
H1C4—C4—C5—H2C5	−64.6	N2—C18—C23—C24	−1.4 (8)
H2C4—C4—C5—C6	−65.6	C19—C18—C23—C22	−1.6 (8)
H2C4—C4—C5—H1C5	174.2	C19—C18—C23—C24	−178.5 (5)
H2C4—C4—C5—H2C5	54.6	C18—C19—C20—C21	0.8 (9)
C4—C5—C6—C1	−61.8 (6)	C18—C19—C20—HC20	−179.2
C4—C5—C6—C7	170.6 (5)	HC19—C19—C20—C21	−179.2
C4—C5—C6—HC6	54.0	HC19—C19—C20—HC20	0.8
H1C5—C5—C6—C1	58.5	C19—C20—C21—C22	−2.2 (9)
H1C5—C5—C6—C7	−69.2	C19—C20—C21—HC21	177.8
H1C5—C5—C6—HC6	174.2	HC20—C20—C21—C22	177.8
H2C5—C5—C6—C1	178.0	HC20—C20—C21—HC21	−2.2
H2C5—C5—C6—C7	50.4	C20—C21—C22—C23	1.6 (9)
H2C5—C5—C6—HC6	−66.2	C20—C21—C22—HC22	−178.4
C1—C6—C7—N2	167.5 (4)	HC21—C21—C22—C23	−178.4
C1—C6—C7—C8	−10.7 (7)	HC21—C21—C22—HC22	1.6
C5—C6—C7—N2	−65.3 (6)	C21—C22—C23—C18	0.3 (8)
C5—C6—C7—C8	116.4 (6)	C21—C22—C23—C24	177.0 (5)
HC6—C6—C7—N2	51.3	HC22—C22—C23—C18	−179.7
HC6—C6—C7—C8	−127.0	HC22—C22—C23—C24	−3.0
N2—C7—C8—C9	−178.1 (5)	C18—C23—C24—C8	2.5 (8)
N2—C7—C8—C24	−1.4 (8)	C18—C23—C24—HC24	−177.5
C6—C7—C8—C9	−0.1 (8)	C22—C23—C24—C8	−174.4 (5)
C6—C7—C8—C24	176.6 (5)	C22—C23—C24—HC24	5.6
C7—C8—C9—C10	−20.7 (7)		
